# Familial amyloidotic polyneuropathy associated with the transthyretin CYS 114 gene in a Russian pair of monozygotic twins.

**DOI:** 10.1186/1750-1172-10-S1-P16

**Published:** 2015-11-02

**Authors:** Igor Srokov Igor, Galina Diukova, Aleksandr Pogromov, Maria Kovalchuk, Eduard Generozov

**Affiliations:** 1 First Moscow Medical University named after I.M. Sechenov, Department of neurological disorders, 119991, Moscow, Russia; 2 University Medical Center, Department of neuro-muscular disorders, 3584 CX, Utrecht, Netherlands; 3 Institute of physical and chemical medicine, Department of genetics, 119435, Moscow, Russia

## Background

Discordant course of the disease in monozygotic (MZ) twins is known to be characteristic for familial amyloidotic polyneuropathy (FAP). Existing cases of FAP in MZ twins refer to amyloidosis due to mutant transthyretin (TTR) Val30Met gene. We present a case of a pair of MZ twins associated with a substitution of tyrosine to cysteine at position 114 in the TTR gene in a Russian kindered. Until now FAP due to mutant TTR Cys 114 has only been described in one Japanese and in one Dutch family. Though in none of them MZ twins were present.

## Materials

Complete laboratory and instrumental investigation of both of the Russian MZ twins was performed. Detailed life history was evaluated in each of the brothers and compared with other known cases of FAP in MZ twins.

## Results

One of the twins had a prominent clinical picture of FAP and visceral amyloidosis, starting around the age of 45 years. In the mean time the other brother was still clinically healthy at the age of 50. DNA confirmed identical mutation of TTR gene in both brothers. Amyloid depositions were found to be similar in the intestines, but not in other locations. Both patients lived in same district and had similar educational background. Though the patient with a prominent clinical picture of FAP experienced vaccination agravated by side effects as well as appendicitis agravated by severe peritonitis in his twenties.

## Conclusion

Charactristic feature of FAP in known pairs of MZ twins is the discordance in the disease course with a prominent manifestation in one of the twins, and delayed disease onset and/or only slight presentation in the other. Genetical and non-genetical factors, or their combination, were supposed to be contributing. Non-genetical mechanisms of the phenotypic variability of FAP could consist of influences on the mutant gene expression during twinning process or along the life. In Russian pair of MZ twins different life-course events could determine clinical presentation of the disease.

